# Supplementary material for: Three months use of Hybrid Closed Loop Systems improves glycated hemoglobin levels in adolescents and children with type 1 diabetes: A meta-analysis
Source: PLoS One. 2024 Aug 12;19(8):e0308202. doi: 10.1371/journal.pone.0308202 (PMC11318905; doi:10.1371/journal.pone.0308202)
Supplement: S1 File — (DOC) [file pone.0308202.s001.doc]

**S1 File.** Search strategies for this study.

**Database**

Medline (Pubmed)

(Pediatrics[MeSH Terms]) OR (Child[MeSH Terms]) OR (Adolescent[MeSH Terms]) OR (Children[Title/Abstract]) OR (Adolescents[Title/Abstract]) OR (Adolescence[Title/Abstract]) OR (Teens[Title/Abstract]) OR (Teen[Title/Abstract]) OR (Teenagers[Title/Abstract]) OR (Teenager[Title/Abstract]) OR (Youth[Title/Abstract]) OR (Youths[Title/Abstract]) OR (Adolescents, Female[Title/Abstract]) OR (Adolescent, Female[Title/Abstract]) OR (Female Adolescent[Title/Abstract]) OR (Female Adolescents[Title/Abstract]) OR (Adolescents, Male[Title/Abstract]) OR (Adolescent, Male[Title/Abstract]) OR (Male Adolescent[Title/Abstract]) OR (Male Adolescents[Title/Abstract]) AND (Insulin Infusion Systems[MeSH Terms]) OR (closed-loop[Title/Abstract]) OR (Infusion System, Insulin[Title/Abstract]) OR (Infusion Systems, Insulin[Title/Abstract]) OR (Insulin Infusion System[Title/Abstract]) OR (System, Insulin Infusion[Title/Abstract]) OR (Systems, Insulin Infusion[Title/Abstract]) OR (Implantable Programmable Insulin Pump[Title/Abstract]) OR (Programmable Implantable Insulin Pump[Title/Abstract]) OR (Pump, Programmable Implantable Insulin[Title/Abstract]) OR (Insulin Pump, Programmable Implantable[Title/Abstract]) OR (Pancreas, Artificial Endocrine[Title/Abstract]) OR (Artificial Endocrine Pancreas[Title/Abstract]) OR (Endocrine Pancreas, Artificial[Title/Abstract]) OR (beta Cell, Artificial[Title/Abstract]) OR (Artificial beta Cell[Title/Abstract]) OR (Artificial beta Cells[Title/Abstract]) OR (Cell, Artificial beta[Title/Abstract]) OR (Cells, Artificial beta[Title/Abstract]) OR (beta Cells, Artificial[Title/Abstract]) AND (Diabetes Mellitus, Type 1[MeSH Terms]) OR (Diabetes Mellitus, Insulin-Dependent[Title/Abstract]) OR (Diabetes Mellitus, Insulin Dependent[Title/Abstract]) OR (Insulin-Dependent Diabetes Mellitus[Title/Abstract]) OR (Diabetes Mellitus, Juvenile-Onset[Title/Abstract]) OR (Diabetes Mellitus, Juvenile Onset[Title/Abstract]) OR (Juvenile-Onset Diabetes Mellitus[Title/Abstract]) OR (IDDM[Title/Abstract]) OR (Juvenile-Onset Diabetes[Title/Abstract]) OR (Diabetes, Juvenile-Onset[Title/Abstract]) OR (Juvenile Onset Diabetes[Title/Abstract]) OR (Diabetes Mellitus, Sudden-Onset[Title/Abstract])) OR (Diabetes Mellitus, Sudden Onset[Title/Abstract]) OR (Sudden-Onset Diabetes Mellitus[Title/Abstract]) OR (Type 1 Diabetes Mellitus[Title/Abstract]) OR (Diabetes Mellitus, Insulin-Dependent, 1[Title/Abstract]) OR (Insulin-Dependent Diabetes Mellitus 1[Title/Abstract]) OR (Insulin Dependent Diabetes Mellitus 1[Title/Abstract])) OR (Type 1 Diabetes[Title/Abstract]) OR (Diabetes, Type 1[Title/Abstract]) OR (Diabetes Mellitus, Type I[Title/Abstract]) OR (Diabetes, Autoimmune[Title/Abstract]) OR (Autoimmune Diabetes[Title/Abstract]) OR (Diabetes Mellitus, Brittle[Title/Abstract]) OR (Brittle Diabetes Mellitus[Title/Abstract]) OR (Diabetes Mellitus, Ketosis-Prone[Title/Abstract]) OR (Diabetes Mellitus, Ketosis Prone[Title/Abstract]) OR (Ketosis-Prone Diabetes Mellitus[Title/Abstract]))) AND (randomized controlled trial[Publication Type]) OR (controlled clinical trial[Publication Type]) OR (randomized[Title/Abstract]) OR (placebo[Title/Abstract]) OR (drug therapy[Title/Abstract]) OR (randomly[Title/Abstract]) OR (trial[Title/Abstract]) OR (groups[Title/Abstract])

**Database**

EMBASE (Ovid SP)

**Search filter**

#1 'Pediatrics'/exp

#2 'Child':ab,ti OR 'Adolescent':ab,ti OR 'Children':ab,ti OR 'Adolescents':ab,ti OR 'Teens':ab,ti OR 'Teen':ab,ti OR 'Teenagers':ab,ti OR 'Teenager':ab,ti OR 'Youth':ab,ti OR 'Youths':ab,ti OR 'Adolescents, Female':ab,ti OR 'Adolescent, Female':ab,ti OR 'Female Adolescent':ab,ti OR 'Female Adolescents':ab,ti OR 'Adolescents, Male':ab,ti OR 'Adolescent, Male':ab,ti OR 'Male Adolescent':ab,ti OR 'Male Adolescents':ab,ti

#3 #1 OR #2

#4 'Insulin infusion'/exp

#5 'Insulin Infusion Systems':ab,ti OR 'closed-loop':ab,ti OR 'Infusion System, Insulin':ab,ti OR 'Infusion Systems, Insulin':ab,ti OR 'Insulin Infusion System':ab,ti OR 'System, Insulin Infusion':ab,ti OR 'Systems, Insulin Infusion':ab,ti OR 'Implantable Programmable Insulin Pump':ab,ti OR 'Programmable Implantable Insulin Pump':ab,ti OR 'Pump, Programmable Implantable Insulin':ab,ti OR 'Insulin Pump, Programmable Implantable':ab,ti OR 'Pancreas, Artificial Endocrine':ab,ti OR 'Artificial Endocrine Pancreas':ab,ti OR 'Endocrine Pancreas, Artificial':ab,ti OR 'beta Cell, Artificial':ab,ti OR 'Artificial beta Cell':ab,ti OR 'Artificial beta Cells':ab,ti OR 'Cell, Artificial beta':ab,ti OR 'Cells, Artificial beta':ab,ti OR 'beta Cells, Artificial':ab,ti

#6 #4 OR #5

#7 'insulin dependent diabetes mellitus'/exp

#8 'Diabetes Mellitus, Type 1':ab,ti OR 'Diabetes Mellitus, Insulin-Dependent':ab,ti OR 'Diabetes Mellitus, Insulin Dependent':ab,ti OR 'Insulin-Dependent Diabetes Mellitus':ab,ti OR 'Diabetes Mellitus, Juvenile-Onset':ab,ti OR 'Diabetes Mellitus, Juvenile Onset':ab,ti OR 'Juvenile-Onset Diabetes Mellitus':ab,ti OR 'IDDM':ab,ti OR 'Juvenile-Onset Diabetes':ab,ti OR 'Diabetes, Juvenile-Onset':ab,ti OR 'Juvenile Onset Diabetes':ab,ti OR 'Diabetes Mellitus, Sudden-Onset':ab,ti OR 'Diabetes Mellitus, Sudden Onset':ab,ti OR 'Sudden-Onset Diabetes Mellitus':ab,ti OR 'Type 1 Diabetes Mellitus':ab,ti OR 'Diabetes Mellitus, Insulin-Dependent, 1':ab,ti OR 'Insulin-Dependent Diabetes Mellitus 1':ab,ti OR 'Insulin Dependent Diabetes Mellitus 1':ab,ti OR 'Type 1 Diabetes':ab,ti OR 'Diabetes, Type 1':ab,ti OR 'Diabetes Mellitus, Type I':ab,ti OR 'Diabetes, Autoimmune':ab,ti OR 'Autoimmune Diabetes':ab,ti OR 'Diabetes Mellitus, Brittle':ab,ti OR 'Brittle Diabetes Mellitus':ab,ti OR 'Diabetes Mellitus, Ketosis-Prone':ab,ti OR 'Diabetes Mellitus, Ketosis Prone':ab,ti OR 'Ketosis-Prone Diabetes Mellitus':ab,ti

#9 #7 OR #8

#10 #3 AND #6 AND #9

#11'randomized controlled trial'/exp OR 'controlled clinical trial'/exp OR randomized:ti,ab OR placebo:ti,ab OR 'drug therapy':lnk OR randomly:ti,ab OR trial:ti,ab OR groups:ti,ab

#12 #10 AND #11

**Database**

Cochrane Central Register of Controlled Trials (CENTRAL) in the Cochrane Library

**Search filter**

#1 Pediatrics[mh]

#2 Child[mh]

#3 Adolescent[mh]

#4 Insulin Infusion Systems[mh]

#5 Diabetes Mellitus, Type 1[mh]

#6 Children

#7 Adolescents

#8 Adolescence

#9 Teens

#10 Teen

#11 Teenagers

#12 Teenager

#13 Youth

#14 Youths

#15 Adolescents, Female

#16 Adolescent, Female

#17 Female Adolescent

#18 Female Adolescents

#19 Adolescents, Male

#20 Adolescent, Male

#21 Male Adolescent

#22 Male Adolescents

#23 closed-loop

#24 Infusion System, Insulin

#25 Infusion Systems, Insulin

#26 Insulin Infusion System

#27 System, Insulin Infusion

#28 Systems, Insulin Infusion

#29 Implantable Programmable Insulin Pump

#30 Programmable Implantable Insulin Pump

#31 Pump, Programmable Implantable Insulin

#32 Insulin Pump, Programmable Implantable

#33 Pancreas, Artificial Endocrine

#34 Artificial Endocrine Pancreas

#35 Endocrine Pancreas, Artificial

#36 beta Cell, Artificial

#37 Artificial beta Cell

#38 Artificial beta Cells

#39 Cell, Artificial beta

#40 Cells, Artificial beta

#41 beta Cells, Artificial

#42 Diabetes Mellitus, Insulin-Dependent

#43 Diabetes Mellitus, Insulin Dependent

#44 Insulin-Dependent Diabetes Mellitus

#45 Diabetes Mellitus, Juvenile-Onset

#46 Diabetes Mellitus, Juvenile Onset

#47 Juvenile-Onset Diabetes Mellitus

#48 IDDM

#49 Juvenile-Onset Diabetes

#50 Diabetes, Juvenile-Onset

#51 Juvenile Onset Diabetes

#52 Diabetes Mellitus, Sudden-Onset

#53 Diabetes Mellitus, Sudden Onset

#54 Sudden-Onset Diabetes Mellitus

#55 Type 1 Diabetes Mellitus

#56 Diabetes Mellitus, Insulin-Dependent, 1

#57 Insulin-Dependent Diabetes Mellitus 1

#58 Insulin Dependent Diabetes Mellitus 1

#59 Type 1 Diabetes

#60 Diabetes, Type 1

#61 Diabetes Mellitus, Type I

#62 Diabetes, Autoimmune

#63 Autoimmune Diabetes

#64 Diabetes Mellitus, Brittle

#65 Brittle Diabetes Mellitus

#66 Diabetes Mellitus, Ketosis-Prone

#67 Diabetes Mellitus, Ketosis Prone

#68 Ketosis-Prone Diabetes Mellitus

#69 #1 OR #2 OR #3 OR #6 OR #7 OR #8 OR #9 OR #10 OR #11 OR #12 OR #13 OR #14 OR #15 OR #16 OR #17 OR #18 OR #19 OR #20 OR #21 OR #22

#70 #4 OR #23 OR #24 OR #25 OR #26 OR #27 OR #28 OR #29 OR #30 OR #31 OR #32 OR #33 OR #34 OR #35 OR #36 OR #37 OR #38 OR #39 OR #40 OR #41

#71 #5 OR #42 OR #43 OR #44 OR #45 OR #46 OR #47 OR #48 OR #49 OR #50 OR #51 OR #52 OR #53 OR #54 OR #55 OR #56 OR #57 OR #58 OR #59 OR #60 OR #61 OR #62 OR #63 OR #64 OR #65 OR #66 OR #67 OR #68

#72 #69 AND #70 AND #71
