# Supplementary figures and images for: Three months use of Hybrid Closed Loop Systems improves glycated hemoglobin levels in adolescents and children with type 1 diabetes: A meta-analysis
Source: PLoS One. 2024 Aug 12;19(8):e0308202. doi: 10.1371/journal.pone.0308202 (PMC11318905; doi:10.1371/journal.pone.0308202)

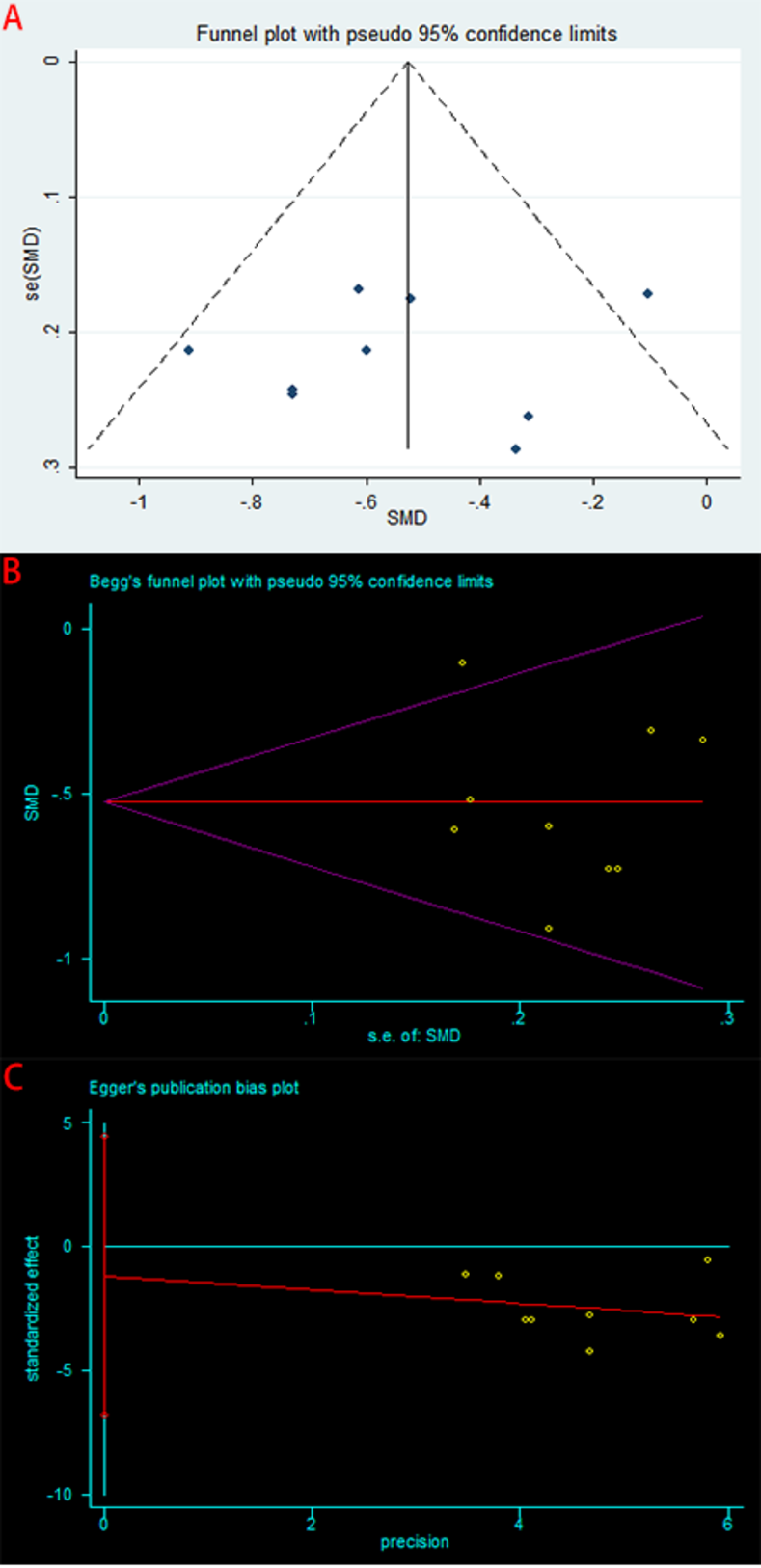

Supplement: S1 Fig — A: A funnel plot of the risk of HbA1c level. B: Begg’s test; C: Egger’s test. (TIF) [file pone.0308202.s003.tif]
