# Supplementary material for: Three months use of Hybrid Closed Loop Systems improves glycated hemoglobin levels in adolescents and children with type 1 diabetes: A meta-analysis
Source: PLoS One. 2024 Aug 12;19(8):e0308202. doi: 10.1371/journal.pone.0308202 (PMC11318905; doi:10.1371/journal.pone.0308202)
Supplement: S1 Table — HbA1c, Glycated hemoglobin level; MD, mean difference; CI, Confidence interval. (DOC) [file pone.0308202.s004.doc]

**S3 Table. Sensitivity analysis for the outcome of HbA1c level, glucose level and percent of sensor glucose values.**

| **Study omitted** | **M-H pooled RR** | |  | **Heterogeneity** | |
| --- | --- | --- | --- | --- | --- |
|  | MD (95%CI) | *P* value |  | I^2^ (%) | *P* value |
| **HbA1c level,%** | **-0.46(-0.62, -0.30)** | **< 0.001** |  | **40** | **0.10** |
| Boughton 2022 | -0.40(-0.53, -0.28) | < 0.001 |  | < 0.001 | 0.53 |
| Elvira Isganaitis 2021 | -0.48(-0.65, -0.31) | < 0.001 |  | 46 | 0.07 |
| H. Thabit 2015 | -0.48(-0.65, -0.31) | < 0.001 |  | 47 | 0.07 |
| J. Ware 2022 | -0.48(-0.68, -0.29) | < 0.001 |  | 47 | 0.07 |
| Julia Ware 2022 | -0.46(-0.65, -0.28) | < 0.001 |  | 47 | 0.06 |
| Lauren G.Kanapka 2021 | -0.45(-0.62, -0.27) | < 0.001 |  | 46 | 0.08 |
| Marc D. Breton 2020 | -0.45(-0.62, -0.27) | < 0.001 |  | 45 | 0.08 |
| Mary B.Abraham 2021 | -0.51(-0.65, -0.36) | < 0.001 |  | 17 | 0.29 |
| R. Paul Wadwa 2023 | -0.46(-0.64, -0.28) | < 0.001 |  | 48 | 0.06 |
| **TIR** | **9.96(7.63, 12.30)** | < 0.001 |  | **51** | **0.04** |
| Boughton 2022 | 9.47(7.15, 11.78) | < 0.001 |  | 47 | 0.06 |
| Elvira Isganaitis 2021 | 9.71(7.24, 12.17) | < 0.001 |  | 53 | 0.04 |
| H. Thabit 2015 | 10.06(7.50, 12.63) | < 0.001 |  | 57 | 0.02 |
| J. Ware 2022 | 10.39(7.46, 13.32) | < 0.001 |  | 56 | 0.02 |
| Julia Ware 2022 | 10.40(7.82, 12.98) | < 0.001 |  | 55 | 0.03 |
| Lauren G.Kanapka 2021 | 9.79(7.26, 12.32) | < 0.001 |  | 55 | 0.03 |
| Marc D. Breton 2020 | 9.78(7.25, 12.31) | < 0.001 |  | 55 | 0.03 |
| Mary B.Abraham 2021 | 10.51(8.65, 12.37) | < 0.001 |  | 13 | 0.33 |
| R. Paul Wadwa 2023 | 9.48(7.08, 11.88) | < 0.001 |  | 48 | 0.06 |
| **< 70 mg/dL** | **-0.22(-0.71, 0.28)** | **0.39** |  | **58** | **0.02** |
| Boughton 2022 | -0.31(-0.70, 0.08) | 0.12 |  | 37 | 0.14 |
| Elvira Isganaitis 2021 | -0.16(-0.76, 0.44) | 0.60 |  | 62 | 0.010 |
| H. Thabit 2015 | -0.16(-0.69, 0.38) | 0.57 |  | 62 | 0.01 |
| J. Ware 2022 | -0.30(-0.83, 0.23) | 0.27 |  | 58 | 0.02 |
| Julia Ware 2022 | -0.25(-0.76, 0.26) | 0.34 |  | 62 | 0.01 |
| Lauren G.Kanapka 2021 | -0.19(-0.79, 0.40) | 0.52 |  | 63 | 0.009 |
| Marc D. Breton 2020 | -0.20(-0.80, 0.40) | 0.52 |  | 63 | 0.009 |
| Mary B.Abraham 2021 | -0.09(-0.49, 0.31) | 0.66 |  | 36 | 0.14 |
| R. Paul Wadwa 2023 | -0.24(-0.80, 0.33) | 0.41 |  | 62 | 0.01 |
| **< 54 mg/dL** | **-0.07(-0.19, 0.06)** | **0.30** |  | **64** | **0.007** |
| Boughton 2022 | -0.07(-0.19, 0.05) | 0.23 |  | 65 | 0.009 |
| Elvira Isganaitis 2021 | -0.08(-0.23, 0.07) | 0.29 |  | 68 | 0.005 |
| H. Thabit 2015 | -0.04(-0.18, 0.09) | 0.55 |  | 63 | 0.01 |
| J. Ware 2022 | -0.09(-0.23, 0.04) | 0.17 |  | 64 | 0.01 |
| Lauren G.Kanapka 2021 | -0.07(-0.22, 0.08) | 0.37 |  | 69 | 0.004 |
| Marc D. Breton 2020 | -0.06(-0.21, 0.09) | 0.42 |  | 68 | 0.004 |
| Mary B.Abraham 2021 | -0.03(-0.12, 0.07) | 0.56 |  | 41 | 0.12 |
| R. Paul Wadwa 2023 | -0.09(-0.23, 0.04) | 0.18 |  | 64 | 0.01 |

HbA1c, Glycated hemoglobin level; MD, mean difference; CI, Confidence interval.

**Continue S3 Table. Sensitivity analysis for HbA1c level, glucose level and percent of sensor glucose values.**

| **Study omitted** | **M-H pooled RR** | |  | **Heterogeneity** | |
| --- | --- | --- | --- | --- | --- |
|  | MD (95%CI) | ***P* value** |  | **I^2^ (%)** | ***P* value** |
| **>180 mg/dL** | **-9.56(-14.17, -4.95)** | **< 0.001** |  | **83** | **< 0.001** |
| Boughton 2022 | -8.24(-12.54, -3.94) | < 0.001 |  | 79 | < 0.001 |
| Elvira Isganaitis 2021 | -9.17(-14.19, -4.15) | < 0.001 |  | 85 | < 0.001 |
| H. Thabit 2015 | -9.70(-14.81, -4.60) | < 0.001 |  | 85 | < 0.001 |
| J. Ware 2022 | -11.09(-14.71, -7.47) | < 0.001 |  | 67 | 0.004 |
| Julia Ware 2022 | -9.78(-14.97, -4.59) | < 0.001 |  | 85 | < 0.001 |
| Lauren G.Kanapka 2021 | -9.28(-14.37, -4.17) | < 0.001 |  | 85 | < 0.001 |
| Marc D. Breton 2020 | -9.28(-14.38, -4.18) | < 0.001 |  | 85 | < 0.001 |
| Mary B.Abraham 2021 | -10.40(-15.50, -5.30) | < 0.001 |  | 83 | < 0.001 |
| R. Paul Wadwa 2023 | -9.13(-14.22, -4.03) | < 0.001 |  | 84 | < 0.001 |
| **>250 mg/dL** | **-6.82(-11.81, -1.83)** | **0.007** |  | **86** | **< 0.001** |
| Elvira Isganaitis 2021 | -6.61(-12.52, -0.71) | 0.03 |  | 89 | < 0.001 |
| Lauren G.Kanapka 2021 | -5.91(-11.39, -0.43) | 0.03 |  | 86 | < 0.001 |
| Marc D. Breton 2020 | -5.90(-11.37, -0.43) | 0.03 |  | 86 | < 0.001 |
| Mary B.Abraham 2021 | -8.73(-11.11, -6.35) | < 0.001 |  | < 0.001 | 0.52 |
| R. Paul Wadwa 2023 | -6.96(-13.44, -0.48) | 0.04 |  | 89 | < 0.001 |
| **>300 mg/dL** | **-2.37(-4.03, -0.70)** | **0.005** |  | **76** | < 0.001 |
| Boughton 2022 | -1.90(-3.54, -0.27) | 0.02 |  | 74 | 0.004 |
| Elvira Isganaitis 2021 | -2.13(-3.89, -0.37) | 0.02 |  | 78 | 0.001 |
| J. Ware 2022 | -2.92(-5.40, -0.44) | 0.02 |  | 79 | < 0.001 |
| Lauren G.Kanapka 2021 | -2.01(-3.74, -0.27) | 0.02 |  | 75 | 0.003 |
| Mary B.Abraham 2021 | -3.12(-4.91, -1.32) | < 0.001 |  | 68 | 0.01 |
| R. Paul Wadwa 2023 | -2.34(-4.29, -0.39) | 0.02 |  | 79 | < 0.001 |
| **Glucose level mg/dL** | **-14.09(-19.48, -8.70)** | **< 0.001** |  | **58** | **0.01** |
| Boughton 2022 | -12.46(-17.18, -7.73) | < 0.001 |  | 44 | 0.08 |
| Elvira Isganaitis 2021 | -13.74(-19.62, -7.85) | < 0.001 |  | 62 | 0.01 |
| H. Thabit 2015 | -14.51(-20.36, -8.66) | < 0.001 |  | 63 | 0.008 |
| J. Ware 2022 | -14.71(-21.60, -7.83) | < 0.001 |  | 63 | 0.008 |
| Julia Ware 2022 | -14.77(-20.58, -8.96) | < 0.001 |  | 62 | 0.01 |
| Lauren G.Kanapka 2021 | -13.82(-19.80, -7.85) | < 0.001 |  | 62 | 0.01 |
| Marc D. Breton 2020 | -13.82(-19.81, -7.83) | < 0.001 |  | 62 | 0.01 |
| Mary B.Abraham 2021 | -15.75(-19.98, -11.53) | < 0.001 |  | 21 | 0.26 |
| R. Paul Wadwa 2023 | -13.38(-19.32, -7.43) | < 0.001 |  | 59 | 0.020 |

MD, mean difference; CI, Confidence interval; Na =Not applicable.
