# Supplementary material for: Three months use of Hybrid Closed Loop Systems improves glycated hemoglobin levels in adolescents and children with type 1 diabetes: A meta-analysis
Source: PLoS One. 2024 Aug 12;19(8):e0308202. doi: 10.1371/journal.pone.0308202 (PMC11318905; doi:10.1371/journal.pone.0308202)
Supplement: S2 Table — HCL, Hybrid Closed Loop Systems. (DOC) [file pone.0308202.s005.doc]

**S5 Table. Subgroup analysis for the outcome of glucose level and percent of sensor glucose values.**

| **Group** | **Number of studies** | **M-H pooled SMD** (standardised mean difference) | | **Heterogeneity** |  |
| --- | --- | --- | --- | --- | --- |
|  |  | **SMD (95%CI)** | ***p*** | **I^2^ (%)** | ***p*** |
| **TIR** | **9** |  |  |  |  |
| **Type of Trial** |  |  |  |  |  |
| Randomized Trial | 7 | 0.805(0.645, 0.965) | < 0.001 | 62.7 | 0.013 |
| Crossover trial | 2 | 1.054(0.755, 1.354) | < 0.001 | < 0.001 | 0.339 |
| **Age(year)** |  |  |  |  |  |
| 0-6 | 2 | 1.146(0.872, 1.420) | < 0.001 | < 0.001 | 0.967 |
| 6-12 | 4 | 0.994(0.750, 1.238) | < 0.001 | < 0.001 | 0.795 |
| 12 -18 | 3 | 0.631(0.238, 1.023) | 0.002 | 65.1 | 0.057 |
| **Therapy Duration of HCL** | |  |  |  |  |
| ≤ 3 m | 2 | 1.028 (0.676, 1.381) | < 0.001 | <0.001 | 0.358 |
| Between 3-6 m | 3 | 1.130(0.883, 1.377) | < 0.001 | <0.001 | 0.996 |
| ≥ 6 m | 4 | 0.696(0.370, 1.021) | < 0.001 | 61.6 | 0.050 |
| **< 70 mg/dL** | **9** |  |  |  |  |
| **Type of Trial** |  |  |  |  |  |
| Randomized Trial | 7 | -0.085(-0.359, 0.189) | 0.543 | 67.3 | 0.005 |
| Crossover trial | 2 | -0.040(-0.487, 0.408) | 0.863 | 50.4 | 0.155 |
| **Age(year)** |  |  |  |  |  |
| 0-6 | 2 | 0.085(-0.170, 0.339) | 0.514 | <0.001 | 0.608 |
| 6-12 | 4 | -0.014(-0.419, 0.391) | 0.945 | 65.9 | 0.032 |
| 12 -18 | 3 | -0.263(-0.689, 0.162) | 0.225 | 71.7 | 0.029 |
| **Therapy Duration of HCL** | |  |  |  |  |
| ≤ 3 m | 2 | -0.116 (-0.449, 0.216) | 0.494 | <0.001 | 0.348 |
| Between 3-6 m | 3 | -0.013(-0.245, 0.219) | 0.911 | <0.001 | 0.426 |
| ≥ 6 m | 4 | -0.069(-0.549, 0.411) | 0.779 | 83.2 | <0.001 |

HCL, Hybrid Closed Loop Systems.

**Continue S5 Table. Subgroup analysis for the outcome of glucose level and percent of sensor glucose values.**

| **Group** | **Number of studies** | | **M-H pooled SMD** (standardised mean difference) | | **Heterogeneity** |  |
| --- | --- | --- | --- | --- | --- | --- |
|  |  |  | **SMD (95%CI)** | ***p*** | **I^2^ (%)** | ***p*** |
| **>180 mg/dL** | **9** |  | |  |  |  |
| **Type of Trial** |  |  | |  |  |  |
| Randomized Trial | 7 | -0.895(-1.226, -0.565) | | <0.001 | 75.6 | <0.001 |
| Crossover trial | 2 | -0.221(-1.0.37, 0.595) | | 0.595 | 83.7 | 0.013 |
| **Age(year)** |  |  | |  |  |  |
| 0-6 | 2 | -0.466(-1.713, 0.781) | | 0.464 | 95.2 | <0.001 |
| 6-12 | 4 | -1.082(-1.329, -0.835) | | <0.001 | <0.001 | 0.405 |
| 12 -18 | 3 | -0.563(-0.993, -0.133) | | 0.01 | 71.2 | 0.031 |
| **Therapy Duration of HCL** | |  | |  |  |  |
| ≤ 3 m | 2 | -0.934 (-1.355, -0.514) | | < 0.001 | 28.2 | 0.238 |
| Between 3-6 m | 3 | -0.658(-1.568, 0.252) | | 0.157 | 92.5 | <0.001 |
| ≥ 6 m | 4 | -0.761(-1.255, -0.267) | | 0.003 | 83.1 | <0.001 |

HCL, Hybrid Closed Loop Systems.
