# Supplementary material for: Three months use of Hybrid Closed Loop Systems improves glycated hemoglobin levels in adolescents and children with type 1 diabetes: A meta-analysis
Source: PLoS One. 2024 Aug 12;19(8):e0308202. doi: 10.1371/journal.pone.0308202 (PMC11318905; doi:10.1371/journal.pone.0308202)
Supplement: S3 Table — HCL, Hybrid Closed Loop Systems. (DOC) [file pone.0308202.s006.doc]

**S6 Table . Meta-regression for the outcome of glucose level and percent of sensor glucose values.**

| **Sources** | **t** | ***P*** | **τ^2^** | **I^2^ Res (%)** | **Adjusted R^2^ (%)** |
| --- | --- | --- | --- | --- | --- |
| **TIR** |  |  |  |  |  |
| Type of Trial | 0.52 | 0.620 | 0.068 | 58.87 | -8.90 |
| Age | -3.25 | 0.014 | < 0.001 | 7.93 | > 0.999 |
| Duration of diabetes | -3.27 | 0.014 | 0.003 | 5.12 | 95.28 |
| Therapy Duration of HCL | -1.70 | 0.134 | 0.035 | 41.73 | 43.99 |
| Jadad Score | -1.42 | 0.198 | 0.047 | 50.56 | 23.69 |
| Year of publication | 0.25 | 0.813 | 0.072 | 63.10 | -15.93 |
| Sample size | -2.14 | 0.070 | 0.020 | 31.46 | 68.43 |
| **<70 mg/dL** |  |  |  |  |  |
| Type of Trial | 0.12 | 0.910 | 0.082 | 65.59 | -21.10 |
| Age | -1.22 | 0.263 | 0.062 | 58.11 | 8.90 |
| Duration of diabetes | -0.99 | 0.356 | 0.067 | 59.65 | 1.43 |
| Therapy Duration of HCL | 0.20 | 0.849 | 0.082 | 66.26 | -21.08 |
| Jadad Score | 0.21 | 0.843 | 0.081 | 66.21 | -19.96 |
| Year of publication | 1.30 | 0.235 | 0.057 | 57.98 | 15.76 |
| Sample size | 0.12 | 0.909 | 0.082 | 65.95 | -21.59 |
| **>180 mg/dL** |  |  |  |  |  |
| Type of Trial | 1.94 | 0.094 | 0.150 | 77.25 | 31.76 |
| Age | -1.10 | 0.922 | 0.256 | 86.41 | -16.57 |
| Duration of diabetes | 0.10 | 0.920 | 0.256 | 86.53 | -16.71 |
| Therapy Duration of HCL | 0.19 | 0.857 | 0.252 | 86.40 | -14.91 |
| Jadad Score | 1.01 | 0.347 | 0.216 | 84.56 | 1.75 |
| Year of publication | -0.02 | 0.988 | 0.252 | 86.47 | -14.80 |
| Sample size | 0.22 | 0.830 | 0.252 | 86.50 | -14.68 |

HCL, Hybrid Closed Loop Systems.
