# Supplementary material for: Three months use of Hybrid Closed Loop Systems improves glycated hemoglobin levels in adolescents and children with type 1 diabetes: A meta-analysis
Source: PLoS One. 2024 Aug 12;19(8):e0308202. doi: 10.1371/journal.pone.0308202 (PMC11318905; doi:10.1371/journal.pone.0308202)
Supplement: Attachment — Submitted filename: detailed response to reviewers.docx [file pone.0308202.s008.docx]

Dear Editor in Chief:

Thanks for your kind suggestions. I have sincerely considered your and the reviewers’ comments.

Thanks again for your time and effort expending on this paper.

Best wishes.

Yours sincerely,

Zhen-feng Zhou

1. Journal requirements:

When submitting your revision, we need you to address these additional requirements.

1.1. Please ensure that your manuscript meets PLOS ONE's style requirements, including those for file naming. The PLOS ONE style templates can be found at

https://journals.plos.org/plosone/s/file?id=wjVg/PLOSOne_formatting_sample_main_body.pdf and

https://journals.plos.org/plosone/s/file?id=ba62/PLOSOne_formatting_sample_title_authors_affiliations.pdf

Answer: we have revised the manuscript according to the PLOS ONE's style requirements.

1.2. Please provide a complete Data Availability Statement in the submission form, ensuring you include all necessary access information or a reason for why you are unable to make your data freely accessible. If your research concerns only data provided within your submission, please write "All data are in the manuscript and/or supporting information files" as your Data Availability Statement.

Answer: we have revised the Data Availability Statement as: “All data are in the manuscript and supporting information files.”

1.3. Please review your reference list to ensure that it is complete and correct. If you have cited papers that have been retracted, please include the rationale for doing so in the manuscript text, or remove these references and replace them with relevant current references. Any changes to the reference list should be mentioned in the rebuttal letter that accompanies your revised manuscript. If you need to cite a retracted article, indicate the article’s retracted status in the References list and also include a citation and full reference for the retraction notice.

Answer: we have reviewed and revised our reference list.

2. Review Comments to the Author

2.1Reviewer #1: The article is good meta-analysis of Closed loop insulin pump use and benefits in the management of Type 1 Diabetes.Extensive references are an asset of any review article. The longterm benefits like A1c reductions and hypoglycaemias are important aspects of a pump use in children & adolescent with Type 1 Diabetes.The total number of subjects in the meta-analysis is good enough to get a tangible and acceptable concusions.

Answer: Thank you so much!

2.2 Reviewer #2: The authors conducted a meta-analysis assessing variations in glycated hemoglobin due to the use of automated insulin delivery systems. The study is methodologically sound, and the results confirm the benefits of these innovative technological devices for diabetes management. However, there is a significant concern regarding the authors' decision to include only randomized clinical trials (RCTs). In the field of diabetes technology, some of the most impactful findings often stem from real-world studies. Can the authors provide a rationale for this choice?

Additionally, there are several typos throughout the text. A thorough revision of the style and language is recommended to enhance the overall quality of the manuscript.

Answer: the purpose of this Meta-Analysis is investigate that 3 months use of HCL could improve glycated hemoglobin levels in adolescents and children with T1D when compared to standard of care. As we know, the best method to compare the advantages and disadvantages of new therapies with standard therapies is a randomized controlled trial, so we only include randomized clinical trials (RCTs). The phenomenon that makes it difficult to interpret the therapeutic effect is called the "confounding effect", and randomization can prevent this effect from occurring. Randomization can ensure that patients participating in different treatment trials have roughly the same physiological characteristics, so there is comparability of data between different groups. If there is a difference in the treatment effect between the two groups at this time, it can be considered that it is caused by the difference in the treatment itself. Therefore, randomization is a crucial step in directly comparing the therapeutic effects of different therapies.

The manuscript was revised by one of the companies that Elsevier recommends for English translation services before we submit it.
