# Supplementary material for: Three months use of Hybrid Closed Loop Systems improves glycated hemoglobin levels in adolescents and children with type 1 diabetes: A meta-analysis
Source: PLoS One. 2024 Aug 12;19(8):e0308202. doi: 10.1371/journal.pone.0308202 (PMC11318905; doi:10.1371/journal.pone.0308202)
Supplement: Attachment — Submitted filename: detailed response to reviewers.docx [file pone.0308202.s009.docx]

Dear Editor in Chief:

I have sincerely considered your and the reviewers’ comments.

We hope that our revised version will be satisfactory for publication in PLOS ONE. Great thanks to you and the referee for the time and effort you expend on this paper.

Best wishes.

Yours sincerely,

Zhen-feng Zhou

1. Journal requirements:

Please review your reference list to ensure that it is complete and correct. If you have cited papers that have been retracted, please include the rationale for doing so in the manuscript text, or remove these references and replace them with relevant current references. Any changes to the reference list should be mentioned in the rebuttal letter that accompanies your revised manuscript. If you need to cite a retracted article, indicate the article’s retracted status in the References list and also include a citation and full reference for the retraction notice.

Answer: we have reviewed and revised our reference list. We remove the reference 43 that have been retracted and delete the discussion of reference 43. Page 12 line 20-23 in Revised Manuscript with Track Changes.

And we replace references 3 with relevant current references.

2.Additional Editor Comments:

Please remove the word "could" from the title as the reviewer has suggested before I can formally accept this manuscript.

Answer: we have removed the word "could" from the title as the reviewer has suggested. Page 1 line 1.

Review Comments to the Author

Dear reviewer, thank you very much for your valuable recommendations. Great thanks to you and the referee for the time and effort you expend on this paper.

Thank you so much, you are so kind!

Reviewer #1: All the quiries of reviewers has been clarified & corrected satisfactorily.The article may be accepted for publication in the latest version.

Answer: Thank you so much, you are so kind!

Reviewer #2: I suggest changing the title. Particularly, removing the word "could" makes the title more impactful.

Answer: thank you very much for your valuable recommendations. We have removed the word "could" from the title as you suggested. Page 1 line 1.
